# An Inquiry into the Difficulties Which Hinder a Complete Registration of Births

**Published:** 1878-09

**Authors:** Henry M. Lyman

**Affiliations:** Professor of Physiology and of Diseases of the Nervous System, Rush Medical College, Chicago


### AN INQUIRY INTO THE DIFFICULTIES WHICH HINDER A COMPLETE REGISTRATION OF BIRTHS.

By Henry M. Lyman. M. D.

(Professor of Physiology and of Diseases of the Nervous System, Rush Medical College, Chicago.)

In the daily papers of this city appeared, not long since, a paragraph, inspired, apparently, by the State Board of Health, in which complaint was entered against the members of the medical profession on account of their neglect of the State law concerning the registration of births and deaths. A similar complaint on the part of the local health officials, last winter, led to the passage of an ordinance by the common council of the city, prescribing the infliction of a heavy fine upon any physician or midwife who should neglect to register the births which might occur in his or her practice.

Now, this disobedience is no new thing under the sun. It is only part and parcel of that indifference to legal requirements which is one of the significant and growing evils of the present time. Everywhere there is an abundance of statute law, and every legislative body at every legislative session, seems impelled, by dire necessity, to add, not its *mite*, but its *quantum* to the existing volume. The lawyers themselves can hardly tell what is law, and what is not—so numerous and so conflicting in some of our communities have the statutes and ordinances become. Under these circumstances, the masses pursue the even tenor of their way, doing about as they please, executing such laws as meet their real wants, and leaving the rest to sink into an oblivion from which they are at length seldom resurrected except for temporary gratification of local or personal malice.

Such being the well-known facts of the case, it seems to be worth while to consider whether this state of things is necessary and irremediable. It would appear to be a reasonable expectation, when any portion of the machinery of State becomes inoperative or fails to satisfy the desires of its authors, that some one should endeavor to search out the causes of such failure. Right here, however, begins one of the chief difficulties in the way of progress. The majority of mankind are destitute of that delineative power which is necessary to every one who would lay before himself a picture of the situation to be examined; consequently they cannot perceive clearly the relations which exist between specified evils and their causes or their effects; and they are therefore incapable of anything better than a slow, blind, empirical search for remedies against the evils of which all are conscious. This same lack of imagination—the faculty which enables its possessor to realize the feelings and the necessities of other people—deadens the majority of people against the sufferings of individuals and classes not their own. Then, again, we are to such an extent creatures of habit, that the majority of mankind would prefer to endure many little ills, and wink at much petty injustice, rather than undertake to effect a change in anything that has once received the sanction of law. This love of routine, combined with lack of imagination, serves also to incapacitate the majority of us for original investigation of the local necessities of a given community with a view to the better adjustment of legislation to those necessities. The sanitary legislation of the country affords a conspicuous illustration of these facts. Instead of studying the peculiar temper and wants of our population, and evolving, in the light of the experience of older States, a system of laws which shall meet those wants, we are much more likely to copy or to compile an abridgement of the sanitary laws of New York or Massachusetts, which in their turn have been borrowed from the laws of England or of Belgium or of Prussia. The consequence of this method is too often the hasty adoption of regulations which, however right and proper they may be in a country where people have been trained for generations to submissive acquiescence with the mandate of an absolute sovereign, are utterly at war with the principles of democratic government and individual freedom. Then, as soon as such ordinances have been transplanted from the soil of despotism in which they first struck root, they soon find shelter under the shadow of bureaucracy which in even the freest governments finds its own interest in the preservation of existing institutions, no matter how illogical or unjust they may be.

Did time permit, I fancy it would be instructive to point out the numerous illustrations of these facts which must occur to every one who thinks. I will only recur to the example with which I commenced this paper—the registration of births. Why is it so difficult to secure what all are agreed should be done? It is done in the countries of Europe. Why not as easily here? Because the organization of society is, in Europe, based on an entirely different foundation from that which exists in America. Even in so free a country as England it is but a short time, comparatively, since the mass o the population was held under the constant surveillance of the established authorities, both civil and religious. It was part of the daily duty of the country squire to know everbody in his vicinity, and to see that every one was about his own business in his own place. It was the duty of the priest to look after all the residents in his parish, making sure that every one paid his tithes, went to confession, took the communion, and had his children safely baptized. Of course it was easy enough in such a community to secure an efficient registry of births and deaths. The baptismal fee was sufficient to make it for the parson's interest to look out for every birth within his reach. Again, in Prussia, the whole nation was organized upon a military basis. Every family was responsible to some official of the government; and for ages it has been an accepted fact that the first duty of every Prussian woman was the production of soldiers for the king. Of course, in such a state of society it seems perfectly natural that the morning report of the nation-camp should contain a return of all the additions to his majesty's forces effected during the previous night. A registry law would only give expression to the precise and artificial habits of a people under the immense pressure of a military despotism.

But when we examine a free and independent society like our own, we find that all the conditions which have made registry laws a success in the old world are still unknown in this country. We have no official class whose existence and privileges are largely dependent upon the care with which they watch the behaviour of the mass of the population. We have over us no military tyrant who must know exactly his resources in men as well as in gold. There is no such thing as a law of entail nor any aristocratic pride of pedigree to be gratified by a long ancestral roll. Consequently, there is only the curiosity of a few scientific men that can be relied upon for the moral support of a registry law. I do not believe that in our city more than a dozen people in every thousand would be found to care for the registration of their nativity even in a family Bible. How shall this difficulty be met? By law, of course. But the law does not get executed, notwithstanding the threat of a penalty for disobedience. Why do not intelligent and generally honorable people, like physicians, obey the law? For two reasons; because they have no personal interest in its execution, and because of an invincible, though perhaps not always clearly recognized feeling of revolt against the essential injustice of the law as it now stands. The present law imposes upon every physician and midwife a special tax in the shape of postage, to say nothing of time occupied in making the returns, and offers no compensation for the extra labor and expense. This is an unjust discrimination against a certain class of citizens.

The commonwealth has the right to demand the services of its citizens, but in no free community has it the right to take such services without compensation. In the words of an eminent English statesmen, "People do not like to make a present to government in any shape or form."

The attempt to impose such a tax is a survival of Asiatic methods of despotism which are only tolerable in an autocratic system of government, where the will of the ruler is sufficient reason for any confiscation which may be practiced upon the property of the subject. It is the instinctive appreciation of this fact which lies at the foundation of the difficulty experienced in securing returns from our physicians. What is the proper remedy? The remedy which naturally suggests itself to the "uncultivated intellect," as one of the greatest of living statesmen styles such a mind, consists in the imposition of a heavy penalty for infraction of the law; and if that fails, an increase of severity is the next resource.

"Tell a person of uncultivated intelligence that any grave crime is increasing, and his first idea will invariably be to increase the severity of the punishment which is assigned to it. Tell him that it is very difficult to get evidence with regard to some particular crime, and the chances are that, if he does not *say*, he will *think* that a little mild judicial torture might have its advantages."—*Nineteenth Century*, May, 1878, p. 811.

But experience—and this method has been tried again and again without success—shows that penalties do not secure obedience. A law which is based upon no public sentiment in its favor, cannot be executed without the previous erection of a vast system of espionage which will never be tolerated in our country. The officials who are now charged with the registration of births might as well try to note the crossing of flies in the air as to follow up all the parturient women in the State. They might occasionally catch and fine an unlucky doctor, but the result would only be an accumulation of indignation against the executors of an unjust law, which would soon effect its repeal.

What, then, is the remedy for this state of things? I do not flatter myself that it is possible to suggest anything which will cover all the necessities created by indolence, carelessness and stupidity on the part of both doctors and officials, but I do believe that something may be done to improve the present situation. The first thing needful is the removal of all unjust discrimination against a particular class of people—the doctors and midwives. It costs postage and envelopes, as well as time, to make these returns. The amount is not large to be sure; but it is as unjust for the State to add fifty cents to a doctor's taxes simply because he is a doctor, as it would be to add fifty dollars. The principle of the thing is the same in either case. It is discrimination against a particular class without a return of corresponding advantages. The State should pay for all such services just as it pays for every other service which is rendered by citizens. It need not incur any great expense—it might, as in the case of jury duty or military service by conscription, fix its own rate of compensation; but the obligation should be recognized, and the principle for which I contend should be preserved. It will not do to attempt the evasion of this argument by saying that the physician is already paid to make these returns, for such is not the fact. The fees of physicians are regulated by long established custom, and any additional charge in the bill would now meet with no more favor than the action of the horse-railway companies when they were allowed by law to collect from each passenger an extra cent to compensate themselves for the tax of a fraction of a cent on each fare. These services are either worth something or they are worth nothing. If they are worth nothing, the infliction of penalties for their refusal is absurd. If they are worth something, they should be paid for by the commonwealth for whose benefit they are rendered. But this would require increased general taxation. Of course it would; and this increase would then be levied on all alike instead of being thrown, as it now is, upon the doctors and midwives alone. Just here lies the whole difficulty in a nut-shell. The health officers are trying to get service from the doctors without paying for it. This was once tried on a considerable scale by our local Board of Health in the matter of sanitary inspection. The matter was finally referred to the courts, and they decided that services rendered to the community must be paid for, and they were accordingly paid for at the rate of one hundred dollars a head. The only difference between the two cases consists in the fact that in one instance the amount at stake was a large one, while in the other it is comparatively a trifle. The principle involved is, however, the same in both cases, and until this is recognized and respected, it will be useless to hope for complete and willing returns of births. Lawmakers who wish to suceed in the enforcement of their laws must first be sure that they are just. Of course, it will be objected to all this by the bureaucrats, that such a scheme is impracticable and cannot be put into execution. In reply to this I would merely say that the official who has not ingenuity sufficient to contrive a method by which all the necessary details of such a plan can be carried into execution without any increase of the present machinery of government, or the necessity for any actual disbursement of money, is not fit to perform the functions of a *legislator*, at least. The whole thing may be done more easily than the taxes are collected. But if, as usual, we must wait for an example from Germany or Holland, we shall have to wait a long time for justice, for, to use the words of the talented author of "Anson's Voyage Around the World," "Human affairs are not always conducted by the plain dictates of common sense. There are many other principles which influence our transactions. And there is one in particular which, though of a very erroneous complexion, is scarcely ever excluded from our most serious deliberations. I mean custom, or the practice of those who have preceded us. This is usually a power too mighty for reason to grapple with; and is the most terrible to those who oppose it, as it has much of superstition in its nature, and pursues all those who question its authority with unrelenting vehemence."

---

We call the attention of the editors of the London *Medical Press and Circular* to the fact that the *Chicago Medical Times* (from which they have made certain excerpts in their number for July 17, 1878, and which they seem disposed to regard as an exponent of regular medicine) is the diminutive organ of the still more diminutive Eclectic sect of medicine men in this city.

